# Dipeptidyl-peptidase-4 (DPP-4) inhibitor ameliorates 5-flurouracil induced intestinal mucositis

**DOI:** 10.1186/s12885-019-6231-y

**Published:** 2019-10-29

**Authors:** Jung Min Lee, In Kyung Yoo, Jae Min Lee, Seung Han Kim, Hyuk Soon Choi, Eun Sun Kim, Bora Keum, Yeon Seok Seo, Yoon Tae Jeen, Hoon Jai Chun, Hong Sik Lee, Soon Ho Um, Chang Duck Kim

**Affiliations:** 1 Division of Gastroenterology and Hepatology, Department of Internal Medicine, Korea University College of Medicine, Seoul, South Korea; 2 Division of Gastroenterology and Hepatology, Department of Internal Medicine, Wonkwang University Sanbon Medical Center, Gunpo, South Korea

**Keywords:** Chemotherapy, Alimentary mucositis, Anti-inflammatory, Fluorouracil, Dipeptidyl-peptidase-4 inhibitor

## Abstract

**Background:**

Chemotherapy-induced alimentary mucositis (AM) is difficult to prevent and treatment is rarely effective. Recent study have been showed that glucagon-like peptide (GLP)-1 and GLP-2 has protective in chemotherapy-induced AM. While the DPP-4 enzyme degrades this GLP-1, the DPP-4 inhibitor blocks the degradation process and raises the concentration of GLP-1. This study aimed to assess the role of DPP-4 inhibitor, a well-known hypoglycemic agent, on chemotherapy-induced AM.

**Methods:**

Twenty-four 6-week-old male C57BL/6 mice were divided into 4 groups: control, 5-fluorouracil (5-FU), DPP-4 inhibitor, and saline (DPP-4i), and DPP-4 inhibitor and 5-FU (DPP-4i + 5-FU). Mucositis was induced by intraperitoneal injection of 5-FU (400 mg/kg). DPP-4 inhibitor (50 mg/kg) was administered orally for four days starting the day before 5-FU administration. Post 72 h of 5-FU injection, mice were sacrificed and body weight change, diarrhea score, villus height, villus/crypt ratio, histologic characteristics including goblet cell count, and mRNA expression of inflammatory cytokines tumor necrosis factor (TNF)-α and interleukin (IL)-6, were assessed.

**Results:**

Daily body weight change was not statistically significant between the 5-FU and the DPP-4i + 5-FU group (*P* = 0.571). Diarrhea score was significantly different between these two groups (*P* = 0.033). In the 5-FU group, the villus height was not maintained well, the epithelial lining was irregular, and inflammatory cell infiltration was observed. Goblet cell count in the DPP-4i + 5-FU group was significantly higher than in the 5-FU group (*P* = 0.007). However, in the DPP-4i + 5-FU group, the villus height, epithelial lining, and crypt structure were better maintained than in the 5-FU group. Compared with the control group, mRNA expression of TNF-α was significantly up-regulated in the 5-FU group. Moreover, mRNA expression of TNF-α in the DPP-4i + 5-FU group was down-regulated compared to the 5-FU group. However, IL-6 in the 5-FU group was significantly down-regulated compared to the control, there was no significant difference in expression of IL-6 between the 5-FU and DPP4i + 5-FU group.

**Conclusion:**

DPP-4 inhibitor can improve 5-FU induced AM and, therefore, has potential as an alternative treatment for chemotherapy-induced AM.

## Background

Chemotherapy-induced alimentary mucositis (AM) is the most common complication in patients receiving chemotherapy [1]. It represents an inflammatory response that lead to severe ulceration, pain, hemorrhage of the gastrointestinal tract and rarely, perforation. In addition, chemotherapy induced AM can promote delay in therapy, prolonged hospitalization, increase the treatment costs and mortality in cancer patients [2]. However, current management of AM is mostly symptomatic treatment including mucosal coating agents, antibiotics, pain control, topical antimicrobials, and cryotherapy [3]. Although the precise mechanism of AM is unclear, the main causes may be reactive oxygen species causing cytotoxic damges to epithelium, tissue, and blood vessels. In addition, up-regulation of pro-inflammatory cytokines such as TNF-α and IL-1β is also one of main mechanism [1].

The dipeptidyl-peptidase-4 (DPP4) inhibitor is a well-known hypoglycemic agent used in patients with type 2 diabetes mellitus. The enzyme DPP-4 degrades the glucagon-like peptide-1 (GLP-1) that is an incretin hormone secreted from intestinal L-cells following food intake. In pancreatic β cells, GLP-1 binds to G protein-coupled receptor (GLP-1R) and stimulates the adenylyl cyclase pathway [4, 5]. Recently, research has shown that GLP-1R is also observed in pancreatic β cells as well as in the gut, lungs, heart, kidney, and central nervous system [6]. Kissow et al. reported that GLP-1 and GLP-2 had an important role in the protection of the small intestine from chemotherapy-induced AM [7]. The mechanism of recovery by which the GLP-1 potentiated insulin-like growth factor-1 receptor signaling seems to be related to beneficial effects in the experimental model of mucositis [8, 9]. Therefore, we hypothesized that the DPP-4 inhibitor via GLP-1 dependent pathway plays an important role in mucosal healing.

However, there was only a few studies that have examined a role of DPP-4 inhibitor in mucositis. In this study, we investigated the potential role of DPP-4 inhibitors in chemotherapy-induced AM of mice models treated with 5-fluorouracil (5-FU).

## Methods

### Animals

Twenty-four 6-week-old male C57BL/6 mice were transported from the Orient Laboratory Animal Company (Seongnam, Korea). Mice acclimated for 14 days. They were kept at a temperature of (22–24 °C), humidity (55%), and 12-h light/dark cycles with individually ventilated cages. The mice were fed Basic diets (LabDiet 5 L79, Orient Laboratory Animal Company, Seongnam, Korea). This animal experiment was approved by the Institutional Animal Care & Use Committee, Korea University (IACUC number: KOREA-2017-0073).

### Experimental protocol and study materials

**Fig. 1:**
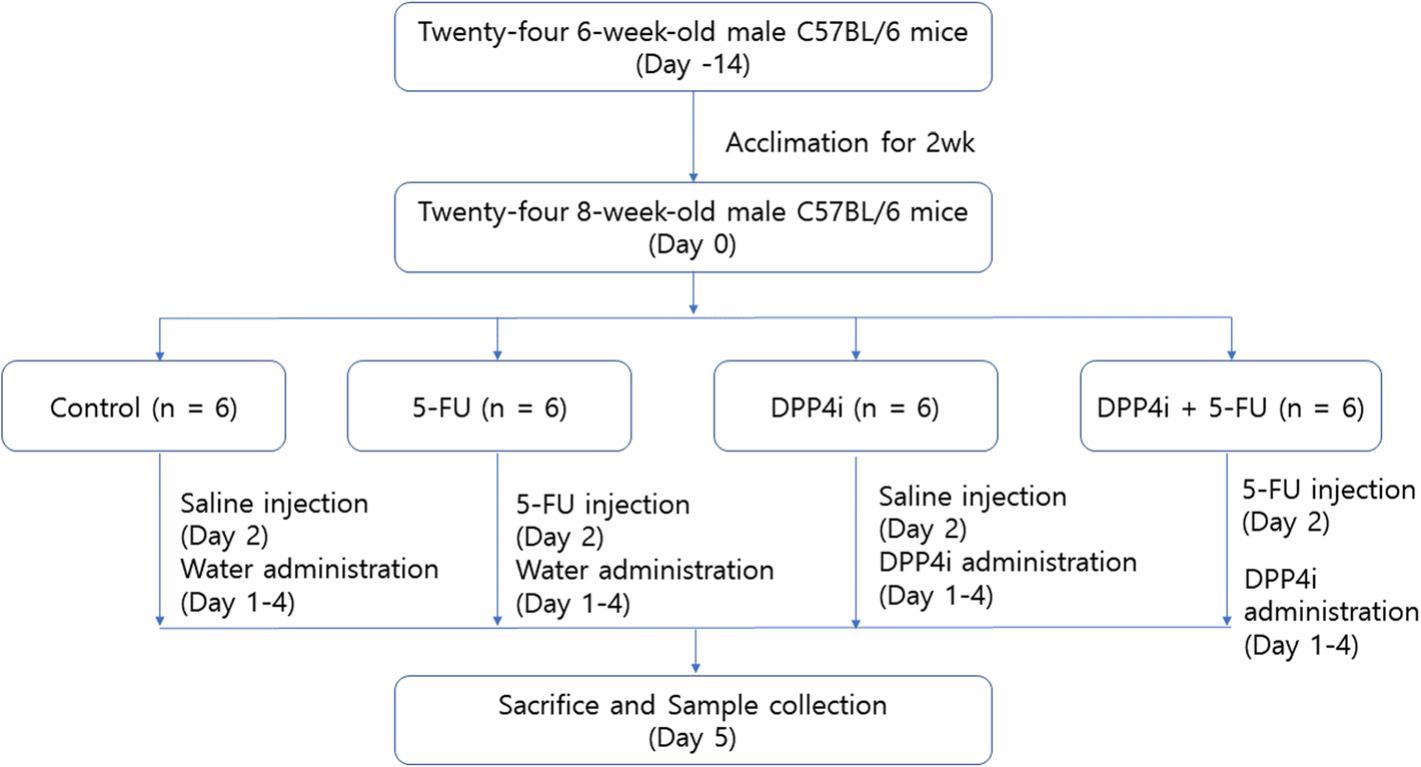
The flow chart of experiment

We divided the mice into four groups: the control group, 5-FU group (5-FU), DPP-4 inhibitor and saline group (DPP-4i), and the DPP-4 inhibitor and 5-FU group (DPP-4i + 5-FU). Mucositis was made with a single intraperitoneal injection of 5-FU (400 mg/kg) (JW Pharmaceutical, Seoul, Korea) [10, 11]. Mice were administered a single intraperitoneal injection of 5-FU into the abdomen at day 2. After that, DPP4 inhibitor 50 mg/kg (Galvus®; Novartis Pharmaceuticals, Nuremberg, Germany) was given orally via gavage once daily from day 1 to day 4. After 72 h of 5-FU injection, the all twenty-four mice were sacrificed and intestinal tissue of the mice was obtained (Fig. 1). All mice were monitored every 12 h for signs of severe mucositis, such as diarrhea, cachexia, bloody stools, or piloerection [7].

### Diarrhea assessment

Based on the Bowen’s score system, the severity of diarrhea was quantified according to stool consistency. 0 = normal stool, 1 = slightly wet and soft stool indicating mild diarrhea, 2 = wet and unformed stool indicating moderate diarrhea, 3 = watery stool indicating severe diarrhea [12].

### Sample collection

Mice were euthanized 72 h after 5-FU injection because 2–3 days have been shown to produce maximal cytokines after 5-FU administration [8–10]. As a method proven in our previous studies [10, 11], mice were euthanized with CO_2_ gas after collecting 1 cc of whole blood from IVC by anesthesia with ketoprofen (5 mg/kg). After we opened the abdomen of mice and obtained small intestines, we cut the jejunum and ileum into 1 cm-long segments and fixed three jejunal specimens and three ileal specimens for 24 h in 10% formalin for histological assessment [7]. For confirming that bone-marrow suppression is sustained by 5-FU, we analyzed white blood cell count (WBC), hemoglobin, and platelet count from whole blood. For cytokine analysis, each three tissue samples were randomly collected from the proximal, middle, and distal thirds of the small intestine, and then snap frozen in liquid nitrogen and stored at − 80 °C.

### Histological assessment

Goblet cells were assessed by the number of goblet cells per 10 villus-crypt units [13]. In a low-power field (40x magnification), villus height and the crypt depth were measured, whereas in a high-power field (200x magnification), villous blunting, fusion, the presence of intraepithelial lymphocytes, and abnormal mitotic forms in the jejunum and ileum were compared among the four groups [7, 13]. Specimens were routinely processed and embedded in paraffin wax, and 4 mm-thick transverse sections were prepared and stained with hematoxylin and eosin (H&E) [10–12].

### cDNA synthesis and quantitative TaqMan PCR

The mRNA expression of interleukin (IL)-6 and tumor necrosis factor alpha (TNF-α) was quantified using TaqMan real-time reverse transcription-polymerase chain reaction (RT-PCR) [14]. cDNA was produced using the ReverTra Ace® qPCR RT Kit* (Code No. FSQ-101). PCR was performed in a QuantStudio Sequence Detection System (Applied Biosystems, Foster City, Calif., U.S.A.) in 384-well microtiter plate using a final volume of 10 ul [15]. After optimum reaction conditions, 2 ul template cDNA was added to the reaction mixture. All samples were amplified on triplicate and data were analyzed with Sequence Detector software (Applied Biosystems) [16].

### Statistical analysis

Statistical analysis was performed using SPSS 20.0 (SPSS Inc., Chicago, IL, USA). Body weight, villus height, crypt depth, and other data were analyzed using a one-way analysis of variance with post hoc test for multiple comparisons. For all measurements, data are expressed as the mean ± the standard error, and differences with a *P* value < 0.05 were considered significant.

## Results

### Body weight change

**Fig. 2:**
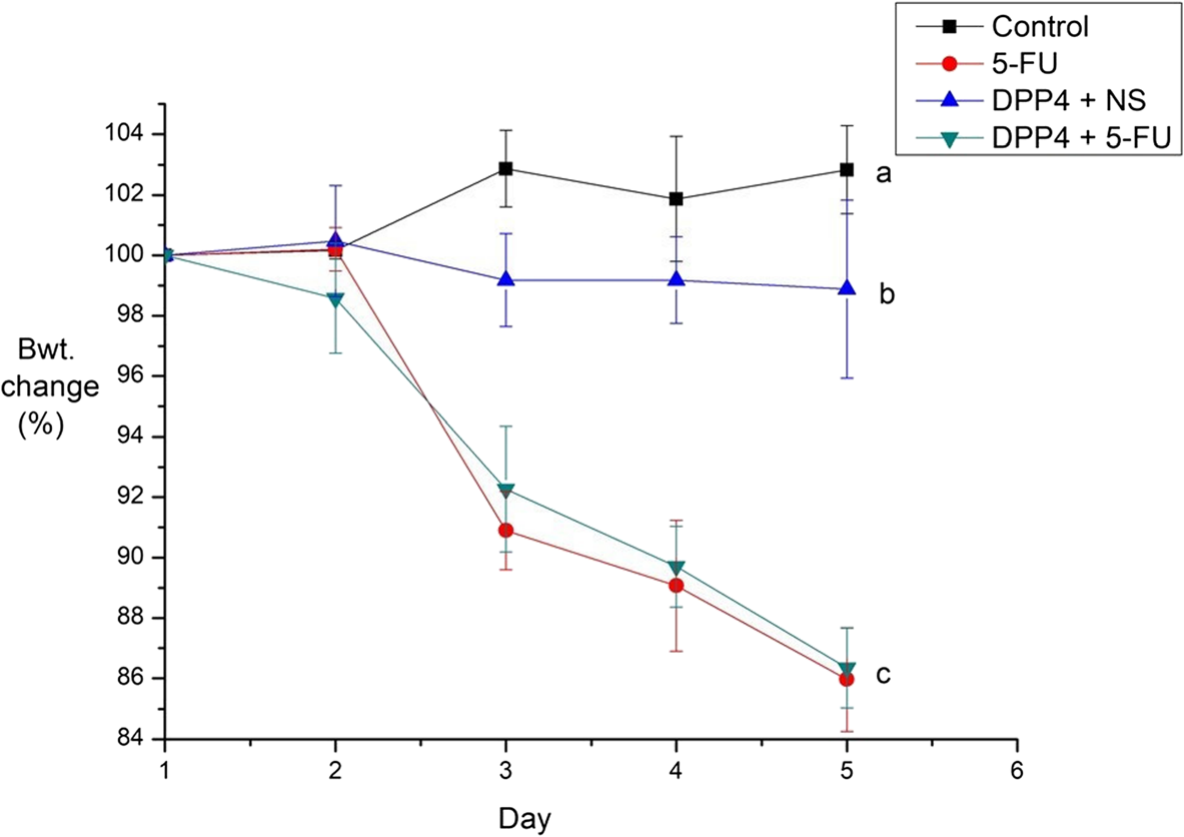
Daily body weight change. The mice in all groups were weighted daily. Body weight as of day 1 is expressed as 100%

During the experiment, all mice were weighed daily. There was no statistically significant difference in body weight during day 1 and 2. After the administration of 5-FU, the control and 5-FU groups had statistically significant weight loss (*P* < 0.001). However, there was no statistically significant difference in body weight between the 5-FU and DPP-4i + 5-FU groups (*P* = 0.571, Fig. 2).

### Diarrhea assessment

**Fig. 3:**
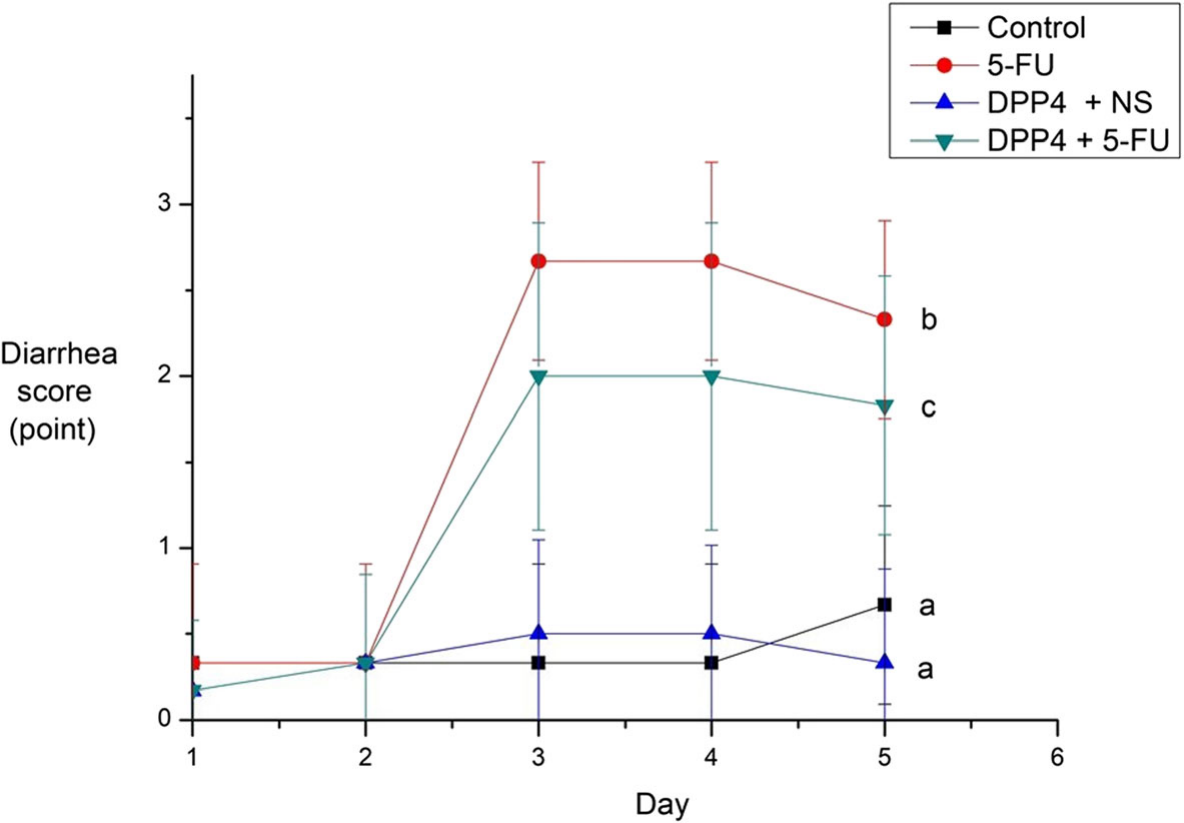
Diarrhea score. Severity of diarrhea in the DPP-4i + 5-FU group was more attenuated than the 5-FU group

Using Bowen’s diarrhea score system, the diarrhea score of the mice was recorded. The saline injection group did not suffer from diarrhea. After 5-FU administration, 5-FU and DPP-4i + 5-FU group showed moderate or more diarrhea on day 3 and improved diarrhea on day 5. There was a statistically significant difference between the 5-FU and the DPP-4i + 5-FU group (*P* = 0.033, Fig. 3).

### Effects of DPP-4 inhibitor on laboratory findings

**Fig. 4:**
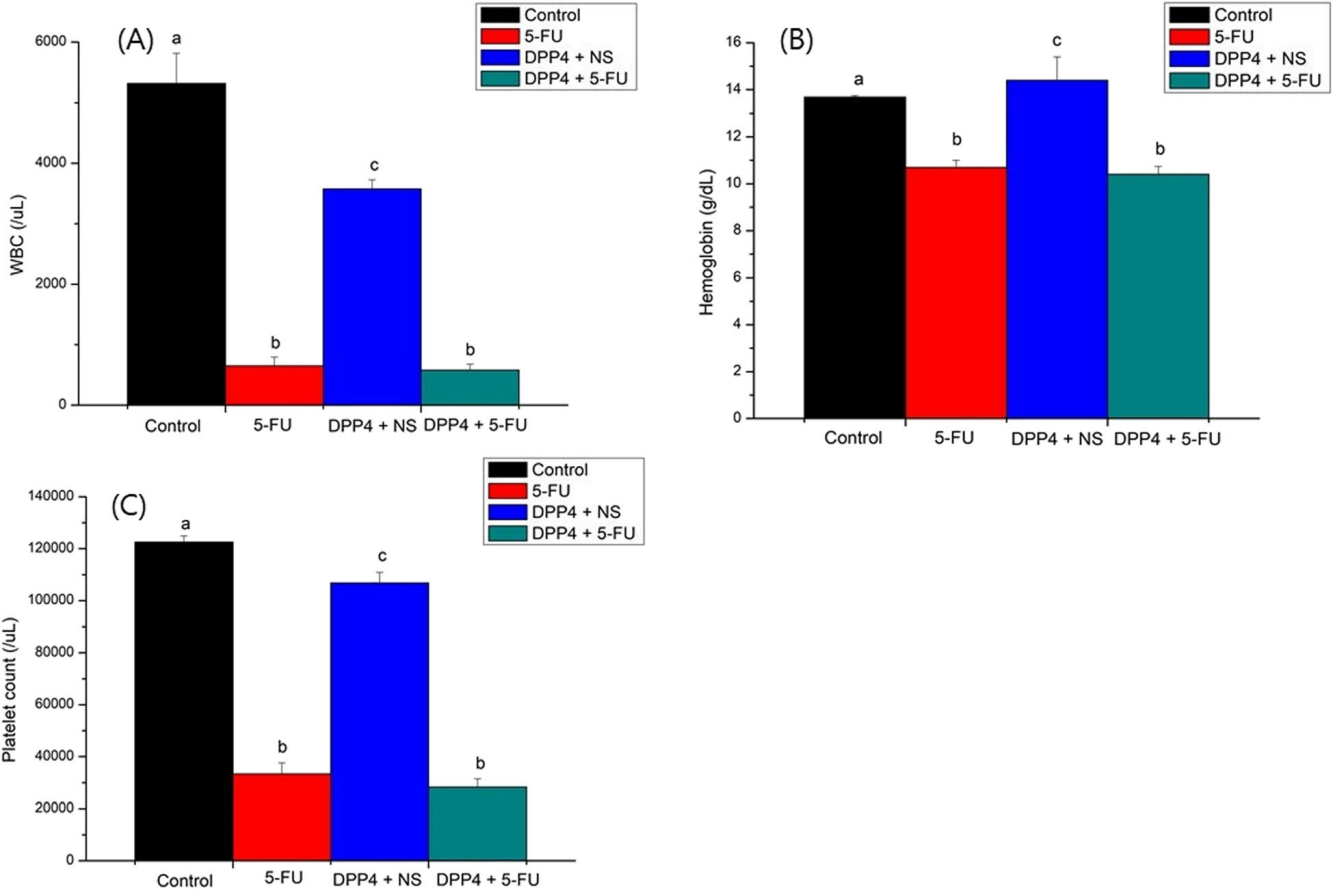
Laboratory findings. WBC, hemoglobin, and platelet counts at the time of sacrifice are shown (**a**) WBC (**b**) hemoglobin (**c**) platelet counts

WBC, hemoglobin, and platelet counts at the time of euthanizing are shown in Fig. 4. The 5-FU and DPP-4i + 5-FU groups had lower WBC count (< 1000 /μL) than the saline group, indicating the effect of 5-FU. However, there was no difference in the WBC count between the 5-FU and the DPP-4i + 5-FU group (*P* = 0.508, Fig. 4a). Hemoglobin levels were also decreased in the 5-FU and DPP-4i + 5-FU groups, but there was no statistically significant difference between the two groups (*P* = 0.155, Fig. 4b). Platelet counts were also reduced to less than 40,000 /μL in the 5-FU group compared to the saline group. However, there was no statistically significant difference between 5-FU and DPP-4i + 5-FU. (*P* = 0.161, Fig. 4c).

### Effects of DPP-4 inhibitor on histologic findings

**Fig. 5:**
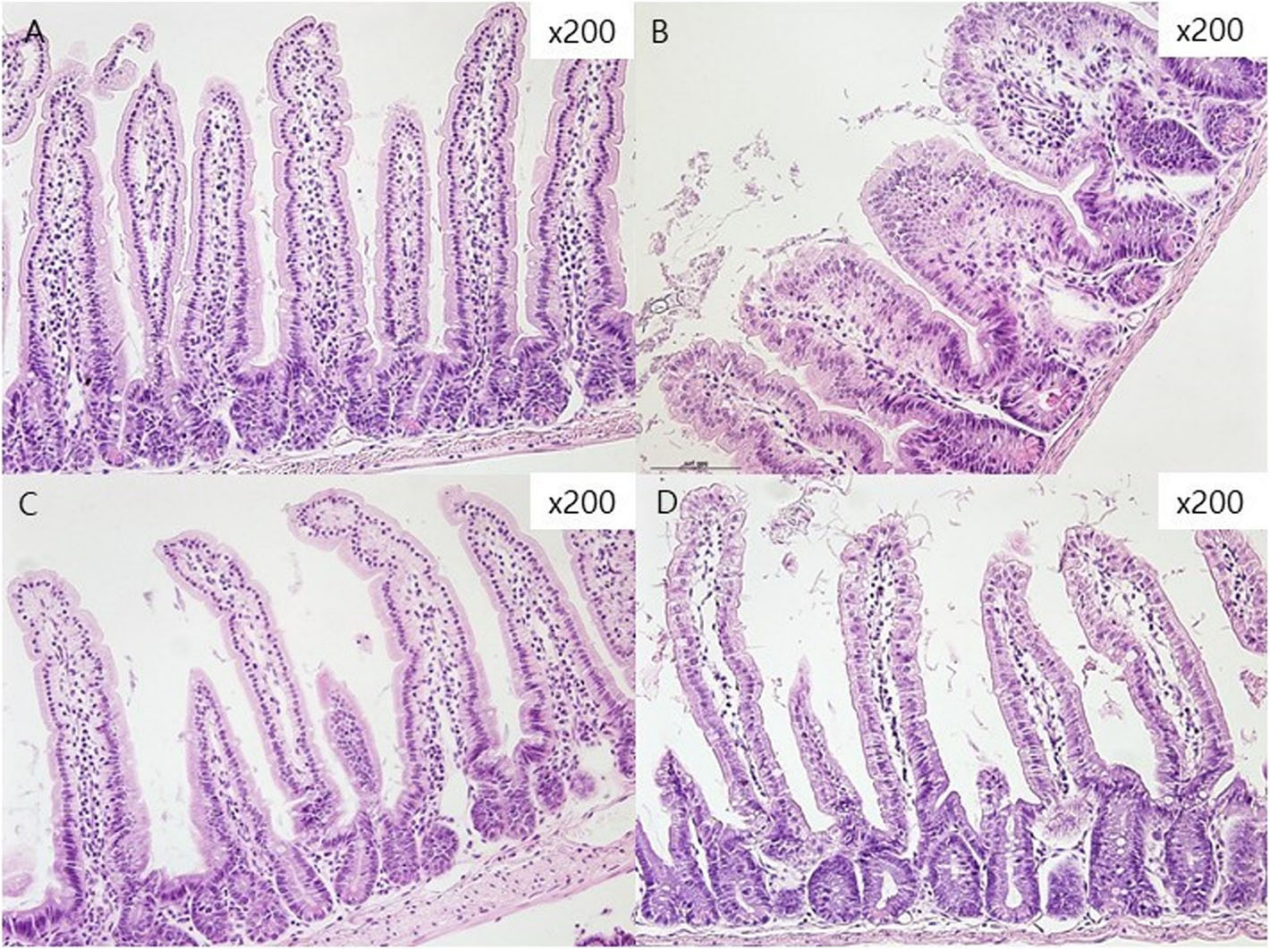
Histological findings of jejunum in mice. **a** control (**b**) 5-Fluorouracil (5-FU) group with significant villus atrophy and crypt dilatation (**c**) DPP-4i group (**d**) DPP-4i plus 5-FU group with less villi destruction and crypt dilatation

**Fig. 6:**
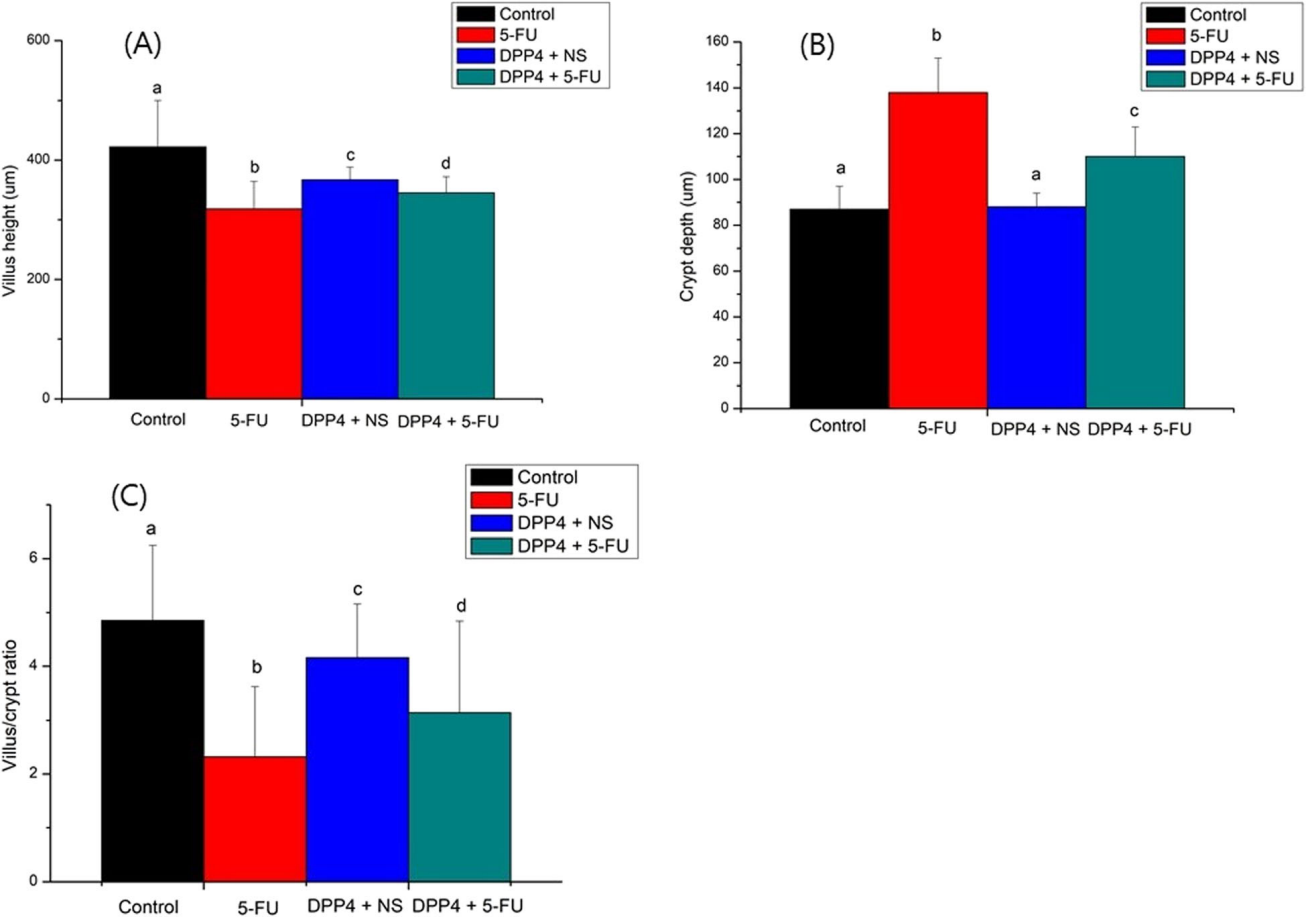
Quantitative value of histologic findings. **a** Villus height (**b**) Crypt depth (**c**) Villus/Crypt ratio

We analyzed the histology to determine the effect of the DPP-4 inhibitor. Figure 5 is a group-by-group comparison of the H & E stain of the jejunum. Mean villous height and crypt depth values are shown in Table 1. In the group treated with 5-FU, the intestinal mucosa showed infiltration of a flattened epithelial layer, shortened villi, and lamina propria with inflammatory cells. The crypt depth was prolonged by cell proliferation in the 5-FU treatment group (Fig. 5). The control group and DPP-4i group had a significantly higher villus height than the 5-FU group and 5-FU + DPP-4i group. Specifically, the villus height in the DPP-4i + 5-FU group was statistically significant compared to the 5-FU group (*P* = 0.007, Fig. 6a). In addition, the crypt depth in the DPP-4i + 5-FU group was also significantly lesser than in the 5-FU group (*P* < 0.001, Fig. 6b). The villus/crypt ratio in the DPP-4i + 5-FU group was also significantly higher than in the 5-FU group (2.32 ± 1.3 vs 3.14 ± 1.7, P < 0.001, Fig. 6c).

**Table 1:** Body weight, diarrhea score, histologic findings, goblet cell count, and laboratory test

	Treatment group			
	Control	DPP-4i	5-FU	5-FU + DPP4i
Body weight change – day 5 (%)	2.83 ± 1.45	−1.12 ± 2.95	*-14.03 ± 1.71	−13.65 ± 1.32
Diarrhea score – day 4 (point)	0.67 ± 0.58	0.50 ± 0.75	*2.33 ± 0.58	‡1.83 ± 0.75
Histologic examination of small intestine				
Villus height (μm)	422.7 ± 78.2	366.9 ± 21.0	*318.2 ± 45.8	‡345.1 ± 27.3
Crypt depth (μm)	87.1 ± 10.2	88.3 ± 5.9	*137.6 ± 15.3	‡109.9 ± 12.7
Villus/crypt ratio	4.85 ± 1.4	4.16 ± 1.0	*2.32 ± 1.3	‡3.14 ± 1.7
Goblet cell count (/field)	6.33 ± 2.08	4.33 ± 0.82	*2.33 ± 0.58	‡4.17 ± 0.75
Lab tests				
WBC (K/uL)	5.32 ± 0.50	3.57 ± 1.53	*0.65 ± 0.15	0.58 ± 0.10
Hb (g/dL)	13.7 ± 0.06	14.4 ± 1.00	*10.7 ± 0.30	10.4 ± 0.34
PLT (M/mm3)	122.6 ± 2.31	106.8 ± 3.97	*33.3 ± 4.22	28.3 ± 3.15

*P < 0.05 vs control, ‡*P* < 0.05 vs 5-FU

**Fig. 7:**
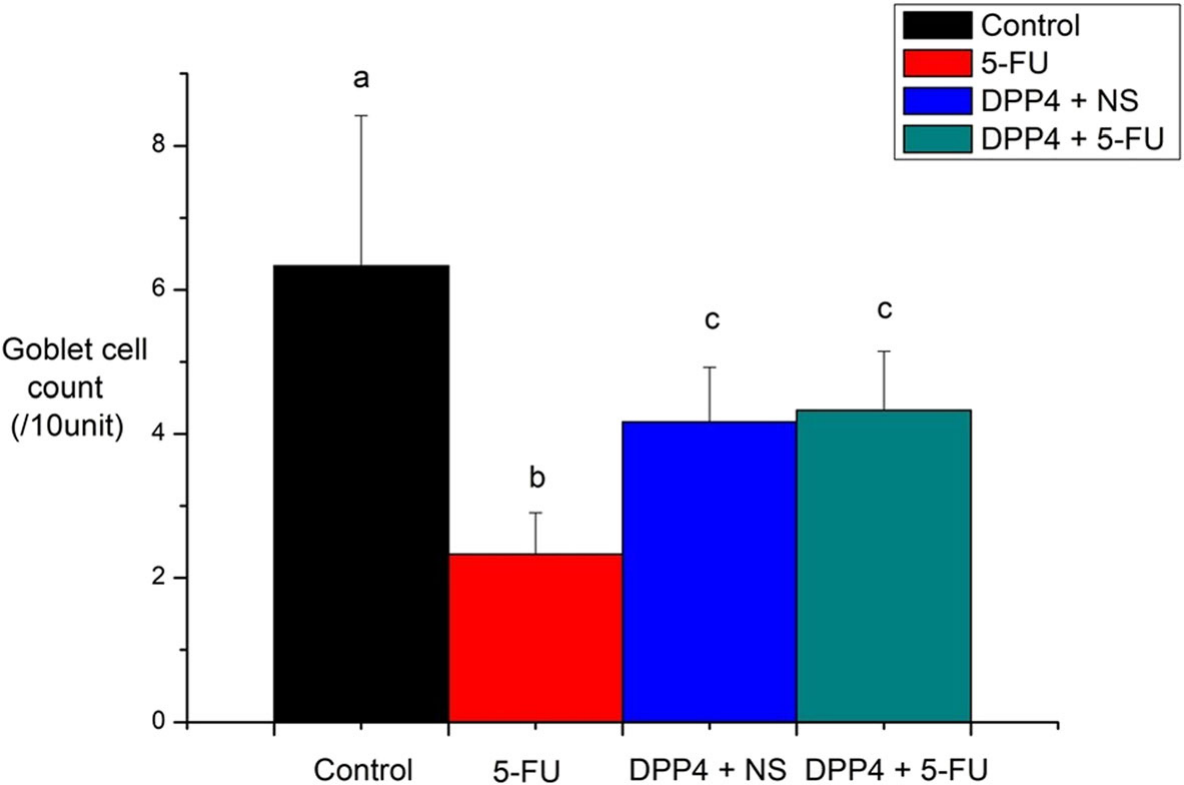
Goblet cell count. Goblet cell count was estimated as number per villus/height unit

Goblet cell staining was calculated as the number per villus/height unit. Similar to other histologic findings, goblet cell count in the 5-FU group was significantly lower than in control group and DPP-4i group (*P* = 0.033, Fig. 7). Goblet cell count in the DPP-4i + 5-FU group was significantly higher than in the 5-FU group (*P* = 0.007).

### Effects of DPP-4 inhibitor on pro-inflammatory cytokine

**Fig. 8:**
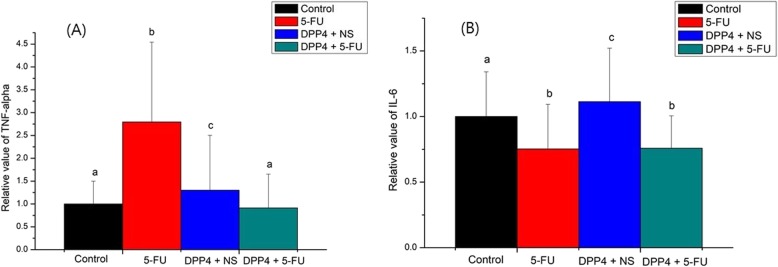
Expression of proinflammatory cytokine. **a** 5-FU group showed significantly higher expression of TNF-α than the control and DPP-4i + 5-FU groups. **b** Expression of IL-6 showed no statistically significant difference between the 5-FU and DPP-4i + 5-FU groups

The mRNA Expression of TNF-α and IL-6 expressed as a fold change for control is shown in Fig. 8. TNF-α was significantly up-regulated in the 5-FU group compared to the control (2.79 vs 1.00, *P* < 0.05). In addition, TNF-α in the DPP-4i + 5-FU group was statistically significantly down-regulated compared to the 5-FU group (2.79 vs 0.91, P < 0.05). Although IL-6 in the 5-FU group was significantly down-regulated compared to the control, there was no significant difference in expression of IL-6 between the 5-FU and DPP4i + 5-FU group.

## Discussion

Chemotherapy-induced AM is one of the common and serious adverse effects of chemotherapy [17]. The exact pathogenesis of chemotherapy-induced AM is unclear but involves cellular damage, generation of reactive oxygen species and stimulation of pro-inflammatory cytokines including TNF-α, IFN-γ, and IL-1β [18–20]. In addition, pro-inflammatory cytokines such as TNF-α, IL-1β and IL-6 are known to play an important role in amplifying the severity of chemotherapy-induced AM [21, 22]. However, the current management of chemotherapy-induced AM is mostly symptomatic treatment and new approaches to prevent and/or treat AM are necessary. In this study, we hypothesized that the DPP-4 inhibitor via GLP-1 dependent pathway plays an important role in mucosal healing. Interestingly, we revealed that DPP-4 inhibitors showed a significant improvement on mice models of 5-FU induced mucositis in diarrhea score, histologic findings, and TNF- α expression. In addition, we found that the DPP-4i + 5-FU group had a more protective effect on especially histopathological findings such as goblet cell count, villus height, and crypt depth against intestinal injury than the 5-FU group.

The DPP-4 inhibitor is a widely used lower glycemic agent for patients with type 2 diabetes mellitus [23, 24]. The enzyme DPP-4 degraded GLP-1, an incretin hormone from intestinal L-cells following food intake [25–27]. When GLP-1 binds to its GLP-1R, it leads to activation in the adenylyl cyclase pathway inducing insulin release. In addition to pancreatic β cells, GLP-1R expression is also observed in multiple organs, including, gut, lungs, heart, kidney, and central nervous system. Recent studies have shown that GLP-1R agonists play a protective role in extra-pancreatic tissue [4, 6]. Yoshiki et al. showed that DPP-4 inhibitors play an anti-inflammatory role in glomerular injury due to reduced macrophage infiltration [4]. In addition, there was a study that provided evidence that after intestinal injury, endogenous GLP-1 and GLP-2 had an important role in the protection of the small intestine from chemotherapy-induced AM [7]. This study showed that endogenous GLP-1 and GLP-2 led to initiation of the healing phase, which was increased after chemotherapy treatment. Although the mechanism of action remains unclear, it appears that GLP-1 promotes the insulin-like growth factor-1 receptor signaling pathway and these growth factors have a beneficial effect in chemotherapy-induced AM [8, 9]. Therefore, we assumed that the DPP-4 inhibitor via the GLP-1 dependent pathway might also have an important role in healing mucositis. However, there was only a few studies providing evidence of a role of DPP-4 inhibitor in mucositis.

We demonstrated that mRNA expression of TNF-α was significantly down-regulated in the DPP-4i + 5-FU group than in the 5-FU group. The results suggest that DPP-4 inhibitors suppressed the proinflammatory cytokine, which might have resulted in the protective effect from 5-FU. In the pro-inflammatory cytokines, TNF-α was significantly down-regulated in the DPP-4i + 5-FU group compared to the 5-FU group, but IL-6 was not significantly down-regulated. This is consistent with our previous study [10].

In this mice model, our results revealed an attenuating effect of DPP-4 inhibitors on the chemotherapy. Our study was the first to investigate the role of the DPP-4 inhibitors in chemotherapy-induced AM and demonstrated a positive effect on the gut as one of the pleiotropic effects of the DPP-4 inhibitor. The mechanism by which the DPP-4 inhibitor improves the effects of AM has not been elucidated yet. However, as previously mentioned, DPP-4 inhibitors may also show reduced macrophage infiltration in chemotherapy-induced AM, as in glomerular injury [4]. In addition, in the pathogenesis of chemotherapy-induced AM, 5-FU and reactive oxygen species cause direct cell damage and NF-*k*B dependent pathways play an important role [7, 28, 29]. In addition, we suggest that the DPP-4 inhibitors play a role in attenuating the severity of mucositis in the TNF-α dependent pathway, as the TNF-α expression is significantly down-regulated in the DPP-4i + 5-FU group. However, further experiments are needed to find out this mechanism.

This study has some limitations. First, we did not perform a dose-dependent study, which might elucidate the efficacy of DPP-4 inhibitors more clearly. In this study, we used a high-dose of DPP-4 inhibitor (50 mg/kg) compared to another reference in a kidney model (10 mg/kg) [4]. Further studies with a low and high dose of DPP-4 inhibitors will be needed. Second, we studied the effects of DPP-4 inhibitors on mainly the small intestine, but not other gastrointestinal tract such as the stomach or colon. Third, although we thought many cytokines to be involved in the pathogenesis of chemotherapy-induced AM, only two of them were measured in this study. We also found that the TNF-α dependent pathway may be an important role in the effect of DPP-4 inhibitors, but further studies are needed to support its mechanism.

## Conclusion

In this study, we revealed that DPP-4 inhibitors showed a significant improvement on mice models of 5-FU induced mucositis in diarrhea score, histologic findings, and TNF- α expression. In conclusion, DPP-4 inhibitors might have a protective effect for intestinal mucositis induced by the administration of 5-FU in mice models.

## Data Availability

The datasets used and/or analyzed during this study are available from the corresponding author on reasonable request. All data generated or analyzed during this study are included in this published article.

## Abbreviations

5-FU: 5-fluorouracil
AM: Alimentary mucositis
DPP-4: Dipeptidyl-peptidase-4
DPP-4i + 5-FU: DPP-4 inhibitor and 5-FU group
DPP-4i: DPP-4 inhibitor and saline group
GLP-1: Glucagon-like peptide-1
GLP-1R: G protein-coupled receptor
H&E: Hematoxylin and eosin
IL: Interleukin
TNF-α: Tumor necrosis factor alpha
WBC: White blood cell count
